# The analytical validation of the Oncotype DX Recurrence Score assay

**DOI:** 10.3332/ecancer.2016.675

**Published:** 2016-09-26

**Authors:** Frederick L Baehner

**Affiliations:** Department of Pathology, University of California, San Francisco, CA, USA and Genomic Health, Inc., Redwood City, CA 94063, USA

**Keywords:** analytical validation, breast cancer, Oncotype DX, prognostic factor, Recurrence Score

## Abstract

*In vitro* diagnostic multivariate index assays are highly complex molecular assays that can provide clinically actionable information regarding the underlying tumour biology and facilitate personalised treatment. These assays are only useful in clinical practice if all of the following are established: analytical validation (i.e., how accurately/reliably the assay measures the molecular characteristics), clinical validation (i.e., how consistently/accurately the test detects/predicts the outcomes of interest), and clinical utility (i.e., how likely the test is to significantly improve patient outcomes). In considering the use of these assays, clinicians often focus primarily on the clinical validity/utility; however, the analytical validity of an assay (e.g., its accuracy, reproducibility, and standardisation) should also be evaluated and carefully considered. This review focuses on the rigorous analytical validation and performance of the Oncotype DX^®^ Breast Cancer Assay, which is performed at the Central Clinical Reference Laboratory of Genomic Health, Inc. The assay process includes tumour tissue enrichment (if needed), RNA extraction, gene expression quantitation (using a gene panel consisting of 16 cancer genes plus 5 reference genes and quantitative real-time RT-PCR), and an automated computer algorithm to produce a Recurrence Score^®^ result (scale: 0–100). This review presents evidence showing that the Recurrence Score result reported for each patient falls within a tight clinically relevant confidence interval. Specifically, the review discusses how the development of the assay was designed to optimise assay performance, presents data supporting its analytical validity, and describes the quality control and assurance programmes that ensure optimal test performance over time.

## Introduction

In the current era, highly complex molecular assays known as *in vitro* diagnostic multivariate index assays (IVD MIA) are performed on formalin-fixed paraffin-embedded (FFPE) tumour samples in order to provide clinically actionable information regarding the underlying tumour biology of each individual patient tumour samples. This actionable information originates from pre-specified correlations between tumour biology, characterised through rigorously validated pre-analytical and analytical laboratory processes, and well-annotated patient clinical outcomes. Appropriate correlation between individual tumour biology and clinical outcome has been shown to provide quantitative information regarding the likelihood of recurrence, i.e. prognosis, and the likelihood of treatment response, i.e., prediction. Having this prognostic and predictive information for each individual patient facilitates personalised treatment decision-making.

**Figure 1.**
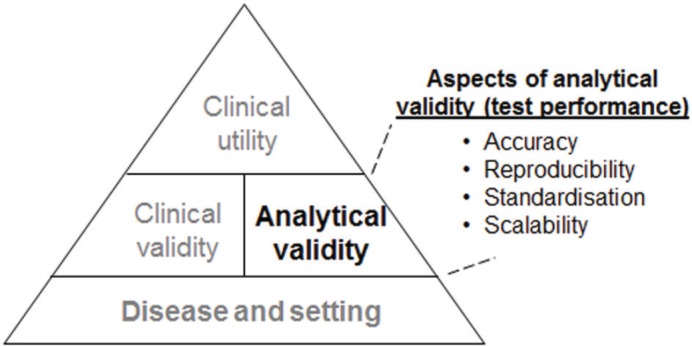
Illustration of the hierarchy of the evidence required for the incorporation of a genomic test into routine clinical practice.

However, these molecular tests are only useful in routine clinical practice if only all of the following are appropriately established: analytical validation (i.e., how accurately and reliably the assay measures the molecular characteristics), clinical validation (i.e., how consistently and accurately the test detects or predicts the intermediate or final outcomes of interest), and clinical utility (i.e., how likely the test is to significantly improve patient outcomes) (Figure 1).

The challenges in establishing analytical validity, clinical validity, and clinical utility are reflected in the huge discrepancy between the number of biomarkers identified in oncology literature (1000s of biomarkers), and the number of biomarkers in clinical use (just over 100 biomarkers) [1]. Unfortunately, often assays do not undergo appropriate analytical validation before they are clinically validated in a pre-specified and blinded manner within a randomised patient trial population.

In considering the use of a molecular assay, clinicians focus primarily on the supporting clinical evidence (i.e., clinical validity and utility). However, the evidence supporting the analytical performance (i.e., analytical validity) of an assay should also be evaluated and carefully considered. Aspects of analytical validity that are relevant to the applicability of the test to patients and ultimately to its adoption into clinical practice include its accuracy, reproducibility, standardisation, and scalability (Figure 1). Notably, even in single-analyte assays such as the routinely-used immunohistochemical (IHC) assessment of oestrogen receptor (ER) or progesterone receptor (PR), the assays present a performance challenge as is reflected in studies demonstrating considerable inter-laboratory variability in IHC-based ER evaluation (especially with respect to detecting ER-positive tumours with low ER positivity) [2–3]. It was also highlighted in the 2010 guidelines by the International Expert Panel convened by the American Society of Clinical Oncology (ASCO^®^) and the College of American Pathologists (CAP) that the published data demonstrated inaccuracies (false positive or false negative) in up to 20% of IHC-based ER and PR status determinations [4]. Challenges to analytical assay performance are accentuated in IVD-MIAs, particularly in assays that require RNA or DNA extraction, amplification, and result determination [5].

This review focuses on the rigorous analytical validation and performance of the Oncotype DX^®^ assay for ER-positive breast cancer. This is a 21-gene (16 cancer genes, 5 reference genes), quantitative real-time reverse-transcriptase polymerase chain reaction (qRT-PCR) assay performed at the Central Clinical Reference Laboratory of Genomic Health, Inc. (Redwood City, CA, USA). After surgical pathologist’s haematoxylin and eosin (H&E) review and marking for tumour tissue enrichment with RNA extraction and gene expression quantitation, then the IVD-MIA uses an automated computer algorithm to produce an individualised patient Recurrence Score^®^ result (a continuous variable ranging between 0 and 100) that has been validated as both a prognostic and predictive tool in patients with ER-positive early-stage breast cancer [6–13]. The review presents evidence showing that the individualised Recurrence Score result reported for each patient falls within a tight clinically relevant confidence interval. Specifically, the review discusses how the development of the assay was designed to optimise assay performance, presents data supporting its analytical validity, and describes the quality control and assurance programmes that ensure optimal test performance.

## Oncotype DX assay development: optimising assay performance

Throughout the developmental stages of the Oncotype DX qRT-PCR platform, laboratory studies were designed to critically assess the optimal analytical performance of the assay platform as well as the choice and optimisation of the RNA extraction chemistry and processes. In addition, studies were conducted to analyse aspects of gene expression variability such as the choice of tissue fixative, within/between FFPE tumour block variability, the impact of contaminants such as biopsy cavities and the optimal tumour tissue input, all of which impact assay performance.

### qRT-PCR platform and the use of RNA extracted from FFPE tumour samples

The technology of the analytical platform chosen for the Oncotype DX assay was real-time qRT-PCR. This technology platform has been shown to be sensitive, specific, and highly reproducible (reviewed by Wong and Medrano [14]). Furthermore, unlike other technologies that use hybridisation-based approaches (e.g., microarrays), real-time qRT-PCR technology is characterised by a wide dynamic range (i.e., it can accurately assess gene expression for genes with very low expression levels as well as for those with very high expression levels) [14–16]. For these reasons, this robust technology platform has been widely deployed globally in molecular pathology laboratories.

RNA derived from FFPE tumour samples was the chosen analyte. This choice permits analysis without disrupting the standard assessment of surgical pathology specimens for the presence of tumour and for critical standard pathological covariates such as tumour size, margins, histology grade, and ER/PR receptor status. Tumour tissue may then be identified for Oncotype DX analysis using expert central surgical pathology review, tumour identification and marking, and tumour enrichment by manual microdissection, after which the enriched tumour tissue undergoes RNA extraction. Technical feasibility studies first investigated the possibility of using RNA extracted from FFPE tumour samples by comparing gene expression profiles obtained from RNA extracted from FFPE tumour sample to those obtained from RNA extracted from fresh-frozen tissue. In these early experiments fresh placenta tissue was used, and the technology used for comparison was the qRT-PCR platform. The gene panel included 42 test genes and 6 reference genes. These proof-of-concept experiments showed that the normalised gene expression profiles were very similar between the two tissue sources (adjusted Pearson correlation, r = 0.91) [17]. RNA extracted from FFPE tumour samples, unlike that extracted from fresh-frozen tissue, is typically characterised by small fragments (less than approximately 300 bases) [17]. However, this observed fragmentation in FFPE tissue-derived RNA occurs at random and therefore does not bias the analysis.

Additional feasibility studies identified the optimal RNA input requirement (375 ng of RNA), established that such a quantity of RNA could be reproducibly extracted from 30 microns of FFPE tissue and demonstrated that more than 50% tumour is required for submission of whole sections (or else, the FFPE tissue must be manually microdissected for tumour tissue enrichment) [18–19].

### Concordance between qRT-PCR and other technologies

Technical feasibility studies compared quantitative mRNA levels of ER, PR, and human epidermal growth factor receptor 2 (HER-2), as assessed by qRT-PCR, with their respective protein levels determined using standard IHC-based assays in 62 breast cancer FFPE tumour specimens. The IHC-based assays were performed by an independent clinical diagnostics laboratory in a blinded fashion. The qRT-PCR and IHC results were highly correlated [17]. These concordance results for ER and PR were later confirmed in two prospective studies. The first was a study of 776 breast cancer patients from the Eastern Cooperative Oncology Group (ECOG) study E2179, showed concordance for ER of 93% and concordance for PR of 88% between central lab IHC and central lab qRT-PCR (Oncotype DX platform) [20]. The second was a study of 568 breast cancer patients from the Kaiser study showed a concordance of 96% for ER and 90% for PR between central lab IHC and central lab qRT-PCR (Oncotype DX platform) [21]. An additional study compared HER-2 evaluation by IHC and fluorescence *in situ* hybridisation (FISH) versus qRT-PCR (Oncotype DX) in 901 patients from the landmark N9831 adjuvant trastuzumab trial in HER-2-positive patients. This study revealed a concordance of 95% between central lab IHC and central lab qRT-PCR, a concordance of 91% between central lab FISH and central lab qRT-PCR, and a concordance of 94% between central lab IHC and central lab FISH [22–23].

All the studies evaluating the expression of ER, PR, and HER-2 using Oncotype DX analysis demonstrated that gene assessment with this platform is highly reproducible [17, 20, 24] and meets the Clinical Laboratory Improvement Amendments of 1988 (CLIA) requirements for proficiency testing (PT) (Genomic Health, data on file, 2015).

### Gene expression variability

Gene expression variability is a key issue that could impact reproducibility of real time qRT-PCR-based assays. Major sources of assay pre-analytical variability are differences in RNA quality and quantity that arise from differences in specimen processing (i.e. delay to fixation, duration of fixation, and differences in fixative). This variability is corrected for by normalisation using five reference genes [6]. Use of multiple reference genes minimises the risk of bias that can result from use of a single reference gene [25]. The reference genes were selected from ten candidate genes, all of which were well-established in the literature as being uniformly and highly expressed across a wide range of breast cancer samples and biological conditions [17]. These were also critically not associated with clinical outcome. The final reference genes were selected because they consistently demonstrated high gene expression and the lowest levels of expression variability between tested patient specimens [17]. The selection of the five reference genes was confirmed in three development studies that were used to refine and finalise the Recurrence Score genes, algorithm, and weight of gene coefficients. In these three studies, the 16 cancer-related genes, and the Recurrence Score results were demonstrated to be consistently associated with clinical outcome (univariate Cox analysis), whereas no such association was observed for the reference genes [6, 26, 27].

Gene expression heterogeneity could potentially also stem from variability within or between tumour blocks. This known source of heterogeneity [28] was investigated in a technical feasibility study in which tumours from two patients with poorly-differentiated breast cancer were examined. The rationale for choosing this histology was the reported association between high tumour grade, genomic instability, and transcriptional variability. For each patient, reference-normalised gene expression for the 16 Oncotype DX assay cancer genes as well as the Recurrence Score result itself were compared within and between tumour blocks (three blocks per patient). Variability in reference-normalised gene expression within and between blocks was generally small, with a total standard deviation (SD) of only 2.2 Recurrence Score units (on a 100-unit scale) within a patient (i.e., including both sources of variability) [29].

**Figure 2.**
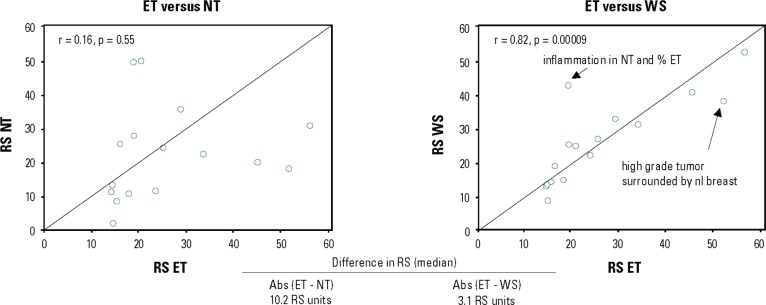
Differences in Recurrence Score (RS) results between enriched tumours (ET), non-tumour (NT), and whole sections (WS). The study included 16 patients with ET, NT, and WS samples (all patients were from the NSABP B-20 Genomic Health study) [19].

Notably, certain aspects of within/between tumour block variability (from the same patient) can be addressed by careful dissection of the tumour sample. In a sub-study of the National Surgical Adjuvant Breast and Bowel Project (NSABP) B-20 Genomic Health Study, the use of manual microdissection to enrich tumour samples was explored. For 31 patients in this study, the amount of invasive cancer was sufficient (≥5% of the section area was invasive cancer) but limited (<70% of the section area), and the sections were suitable for manual microdissection. The study compared gene expression in enriched tumour samples, non-tumour samples, and whole sections from the same patients. The study showed large variability in Recurrence Score results between enriched tumour samples and non-tumour samples (median difference in Recurrence Score results of 10.2 units) and smaller but significant variability between enriched tumour samples and whole samples (median difference in Recurrence Score results of 3.1 units) (Figure 2), suggesting that manual microdissection is appropriate in certain pre-specified cases that contain relatively large amounts of non-tumour elements [19]. A specific aspect that can be addressed through manual micro-dissection is the existence of biopsy cavities in certain FFPE tissue samples, as the presence of these cavities may impact test results and reduce reproducibility. A technical feasibility study explored the impact of having biopsy cavities by comparing (in the same specimens) reference-normalised gene expression for the 16 cancer genes included in the Oncotype DX assay in whole sections of submitted specimens and tumour sections that were enriched by excluding the biopsy cavities using microdissection. Specimens from 48 breast cancer patients with a spectrum of histological grades, ranging from low to high, were included in the analysis. The study revealed statistically significant differences in reference-normalised gene expression for six genes. Also, although overall the concordance between the Recurrence Score result of the whole-section and that of the tumour-enriched section was high (0.92), a statistically significant difference in Recurrence Score results was noted for moderately-differentiated tumours [30]. These findings established that excluding biopsy cavities is essential for precise Recurrence Score assessment. Hence all specimens submitted for Oncotype DX testing have biopsy cavities removed via manual microdissection after histologic review by board certified surgical pathologists (Genomic Health, data on file, 2015).

## Analytical validation of the Oncotype DX assay

The analytical validation process for the Oncotype DX assay included creating standard operating procedures (SOPs) for the entire assay process. These digital SOPs include detailed specifications for the assay and constitute the basis for operation of the reference laboratory. They are updated periodically and reviewed by a multidisciplinary team.

The analytical validation process was performed to ensure that the laboratory procedures used in the generation of the Recurrence Score result are accurate, precise, and reproducible. Thus once all pre-analytical and analytical SOPs were finalised, the certified clinical laboratory assistants used them to perform the clinical validation study as well as to handle clinical laboratory samples in order to ensure process continuity between the clinical validation and clinical laboratory patient results. The validation process that was used was analogous to that used for single-analyte assays. It characterised various aspects of test performance such as amplification efficiency, linearity, dynamic range, reproducibility, and analytical precision all of which are related to the qRT-PCR process that underlies Oncotype DX testing. The validation was performed both for the individual genes within the assay as well as for the Recurrence Score results [18]. The process also defined the specifications for continuous process monitoring and control.

The analytical validation demonstrated performance ranges including consistency in amplification efficiency between genes and linearity over a wide range with an estimated maximum deviation from linearity of <1 cycle threshold (C_T_) units over ≥2000-fold range of RNA concentrations. The mean quantification bias was 0.3% and the coefficients of variation (CVs) were found to be 3.2–5.7%, which is well within the accepted range [18]. Process monitoring in the clinical reference laboratory demonstrated high reproducibility with a cumulative SD (representing all sources of process variation) of <2 Recurrence Score units on a 100-unit scale [18].

## Clinical reference laboratory operation: test performance aspects

### Regulatory standards

Oncotype DX testing is performed in the Genomic Health Clinical Reference Laboratory in the United States which is regulated under CLIA (United States federal regulatory standards that apply to all clinical laboratory testing performed on humans in the United States, except clinical trials and basic research). The Genomic Health, Inc. lab is CLIA-certified for high-complexity clinical testing and is also accredited by CAP (Genomic Health, data on file, 2015). The CAP accreditation programme is based on rigorous quality standards that are reflected in checklist requirements, which are used by inspection teams biennially to assess the management/operation of the laboratory. The CAP accreditation meets and exceeds the regulatory requirements [31]. Audits by CAP are thorough and include assessment of Oncotype DX validation reports, the procedure manual, the quality control, and the quality assurance processes. The laboratory is also inspected biennially by the State of New York Department of Health. The Clinical Laboratory Evaluation Programme (CLEP) seeks to ensure the accuracy and reliability of results of laboratory tests on specimens obtained within the state through on-site inspections, proficiency testing, and evaluation of the qualifications of personnel of state permit-holding clinical laboratories and blood banks. Since the Genomic Health Clinical Reference Laboratory opened in 2004, there have been nine on-site audits with no non-conformities reported (Genomic Health, data on file, 2015).

### Quality systems

Genomic Health has implemented a comprehensive quality assurance programme encompassing all aspects of the operation of the Clinical Reference Laboratory. This programme includes the following elements: a document control process using digital transaction management and electronic signature to ensure that laboratory policies and SOPs are properly reviewed and approved by subject matter experts (including the Laboratory Director) prior to their implementation; a comprehensive training and competency assessment programme for laboratory staff which ensures that the staff is appropriately trained to perform the required testing and that the competency of the staff is assessed periodically; a proficiency testing programme ensuring that the accuracy and reliability of analytical results are assessed regularly; a corrective and preventative action process ensuring that deviations, errors, and complaints are investigated and resolved; regular audits of the areas in which the specimens are received and accessioned i.e. monitoring specimen collection, labelling, transport and storage processes; and quarterly compilation of key performance indicators by a cross-functional team including the Laboratory Director (these indicators are reviewed at quarterly quality assurance and improvement meetings to ensure that quality is maintained and to identify relevant trends) (Genomic Health, data on file, 2015).

### Standardisation and monitoring of patients’ samples

**Figure 3.**
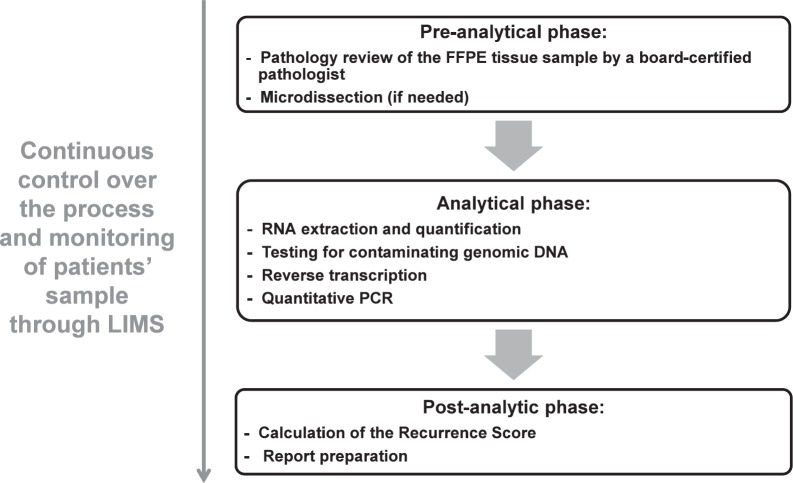
Oncotype DX assay process steps. Abbreviations: FFPE, formalin-fixed paraffin embedded; LIMS, Laboratory Information Management System; PCR, polymerase-chain reaction.

The testing process includes a pre-analytical phase (histological and pathological), an analytical phase (clinical laboratory), and a post-analytical phase (information technology [IT] result generation). All aspects of these phases including reagents, liquid-handling robots, analytical instruments, and methods are standardised and rigorously controlled (Figure 3) (Genomic Health, data on file, 2015).

A key element to this control is the Laboratory Information Management System (LIMS) that integrates control over the process with tracking of all qualified laboratory reagents/supplies and patient samples through barcoding. LIMS barcoding enables traceability of the history of each patient’s sample as it moves through the entire laboratory testing process. The LIMS also tracks the reagent lots, liquid handling robots, analytical instruments, as well as dates, times, and operators at the various process steps thereby allowing monitoring of test results generated in each of the process steps. In-process checks are used to verify the identity, quantity, and expiration date of the reagents used in each processing step. Another key component of standardisation is reagent quality control. To this end, in the Genomic Health Clinical Reference Laboratory, all critical reagents used in the testing process are inspected and tested (with very stringent specifications) as appropriate to ensure their suitability for use in patient sample testing. Each shipment of incoming raw materials is checked for conformance with the stringent quality specifications of the laboratory using various analytical and functional test methods. The quality specifications set forth by the laboratory are often far more stringent than those of the manufacturers and are specifically designed to ensure accuracy and reproducibility of the Oncotype DX assay results.

Standardisation is maintained throughout the testing process. The standardisation of the pre-analytical phase begins with the pathologist at the local site following the Oncotype DX pathology guidelines (i.e. choosing a tumour block with the greatest amount/area of the highest grade carcinoma that is morphologically consistent with the submitting diagnosis) (Genomic Health website, 2015). The standardisation in the pre-analytical phase continues with the arrival of the sample to the Genomic Health Clinical Reference Laboratory. Once in the lab a pathology review of the FFPE tumour sample is performed by a board-certified surgical pathologist with expertise in breast pathology. They determine whether the block contains tumour, if so, the histologic subtype of the tumour and whether manual microdissection is required for tumour enrichment (depends if less than 50% is invasive carcinoma or there is presence of biopsy cavities). In the analytical phase—which includes RNA extraction and quantitation, testing for contaminating genomic DNA, reverse transcription, quantitative PCR and data-quality control—standardisation of RNA extraction is noted to be key, as this step can introduce significant levels of analytical variability/bias into the test results, particularly when the specimens used are paraffin-embedded [28]. Assays with no single standardised RNA extraction method are inherently subject to greater variability than those using a single tightly controlled extraction method. For Oncotype DX testing, standardisation of the RNA that is analysed in the real-time qRT-PCR assays is achieved through using an optimised and validated RNA extraction method that has high yield of high-quality RNA (the method is automated to maximise reproducibility) followed by quantification of the extracted RNA. The RNA is also subjected to an extremely sensitive test for the presence of any contaminating genomic DNA, as the presence of such DNA would bias the results.

The LIMS also facilitates identifying any bias that may arise in any step of the testing process as all the data are centralised (as opposed to decentralised testing where identifying such biases is more challenging). With the huge number of Oncotype DX tests performed thus far (approximately 500,000 from January 2004 through March 2015; Genomic Health, data on file, 2015), Genomic Health through acquired experience has refined its ability to identify and manage biases related to testing.

### Reproducibility

To monitor and control the reproducibility of the assay with respect to each of the real-time qRT-PCR tests being performed as well as the test as a whole, each of the 21 genes in the assay is tested in triplicate. Expression values for each gene are assigned when at least two of the three real-time qRT-PCR reactions provide acceptable results (PCR controls are run in every assay plate and non-acceptable results are identified by their amplification curves). In order for the Recurrence Score result to be calculated, all 21 genes included in the Oncotype DX assay must have an expression value assigned [18]. Also, the assay was designed to have lower SDs when the Recurrence Score results are low. That is because the confidence in the results should be the highest when the testing may result in the patient forgoing adjuvant chemotherapy.

### Reporting

**Figure 4.**
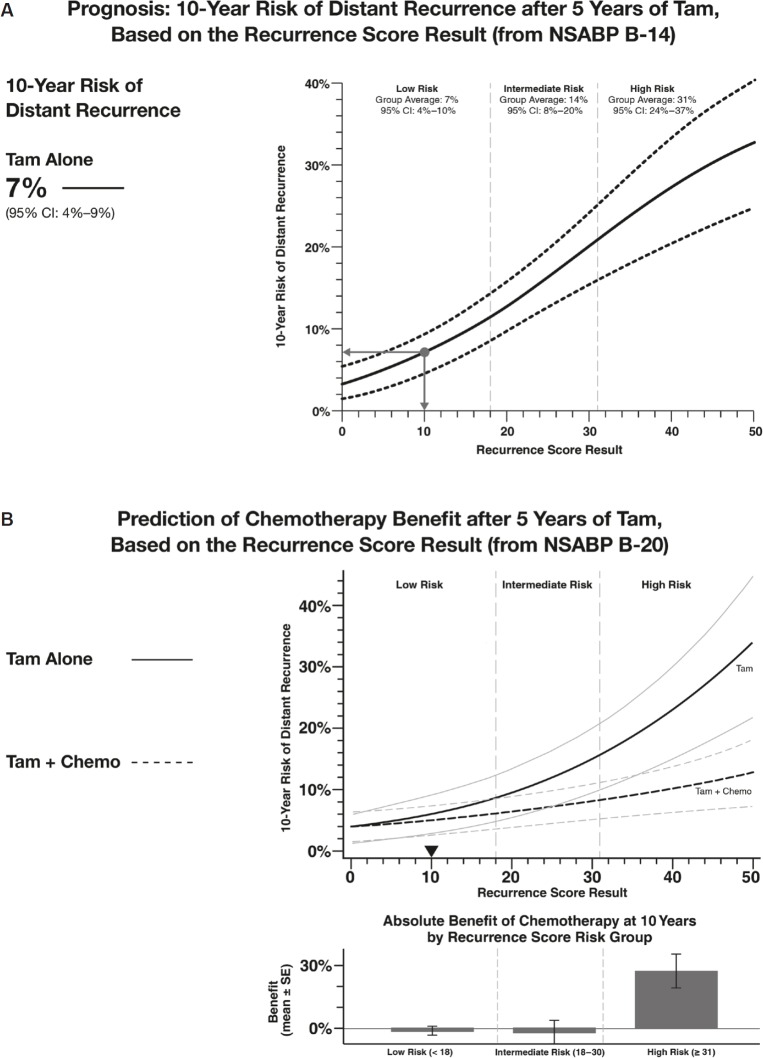
Oncotype DX reports present the Recurrence Score result as a continuous parameter. The figure presents sections of a sample report showing the prognostic (A) and predictive (B) implications of a Recurrence Score result of ten for a hypothetical oestrogen-receptor positive node-negative breast cancer patient. Tam = tamoxifen.

In the postanalytical phase of the testing process, an individualised report is generated for each patient that includes the Recurrence Score result. The report also includes the prognostic and predictive implications of the Recurrence Score result based on the validation studies that are appropriate to that specific patient with respect to their nodal involvement (node negative, 1–3 positive nodes, or ≥4 positive nodes) (Figure 4). Providing the Recurrence Score result as a continuous parameter is reflective of the underlying tumour biology and individualised assessment of distant recurrence risk. The report also includes quantitative single-gene assessments for ER, PR, and HER-2, all of which are also routinely evaluated in the initial pathological evaluation of the tumour.

The routine evaluation of ER and PR status is performed by IHC by using anti-ER and anti-PR antibodies that interact with the ER and PR proteins respectively. This approach became the standard method for determining hormonal receptor status in the 1990s. According to the updated ASCO/CAP guidelines (2010), the pathological report should include the percentage of tumour cells that exhibit nuclear immunostaining, the intensity of the staining, and its interpretation. The interpretation section should state whether the tumour is ER or PR positive (≥1% of tumour cell nuclei are immunostained), negative (<1% of tumour cell nuclei are immunostained and positive intrinsic controls are immunoreactive), or uninterpretable (no immunostained tumour nuclei and no nuclear immunoreactivity in the positive controls) [4].

The Oncotype DX-based assessment of ER and PR is based on mRNA expression levels. This assessment was demonstrated to be concordant with IHC-based assessment in several studies [17, 20]. Thus, the Oncotype DX single-gene reporting of ER and PR provides an alternate method for determining ER and PR status that could complement the IHC-based information, particularly for patients whose ER/PR results are borderline positive or for those whose IHC results were uninterpretable.

The routine evaluation of HER-2 status is performed by IHC (protein levels) or *in-situ* hybridisation (ISH; mRNA levels). According to the updated ASCO/CAP guidelines (2013), HER-2 status could be determined by either method and if the results are equivocal, reflex testing should be performed using the alternate assay (IHC or ISH) [32]. Similar to the ER/PR determinations, the Oncotype DX-based HER-2 assessment is based on mRNA expression levels and was demonstrated to be concordant with the IHC assessment in several studies [17, 24]. Thus as in ER/PR single-gene reporting, the Oncotype DX single-gene reporting of HER-2 provides an alternate method that could complement the IHC/ISH findings, particularly for patients whose HER-2 status was indeterminate. Thus as in ER/PR single-gene reporting, the Oncotype DX single-gene reporting of HER-2 provides an alternate method that could complement the IHC/ISH findings, particularly for patients whose HER-2 status was indeterminate or in order to prompt additional testing by IHC or ISH.

## Conclusions

High performance of genomic tests with respect to accuracy, reproducibility, standardisation and scalability is crucial for ensuring that the test results represent the biological characteristics of the tumour and should be considered in the treatment decision process. The performance of the Oncotype DX assay performed in the Clinical Reference Laboratory of Genomic Health is continuously monitored against the performance of the approximately 500,000 tests performed thus far, thereby ensuring reliable results over time for breast cancer patients and their physicians.
